# Sensing and Modelling Mechanical Response in Large Deformation Indentation of Adherent Cell Using Atomic Force Microscopy

**DOI:** 10.3390/s20061764

**Published:** 2020-03-22

**Authors:** Tianyao Shen, Bijan Shirinzadeh, Yongmin Zhong, Julian Smith, Joshua Pinskier, Mohammadali Ghafarian

**Affiliations:** 1 Robotics and Mechatronics Research Laboratory, Department of Mechanical and Aerospace Engineering, Monash University, Clayton, VIC 3800, Australia; bijan.shirinzadeh@monash.edu (B.S.); joshua.pinskier@monash.edu (J.P.); Mohammadali.Ghafarian@monash.edu (M.G.); 2 School of Engineering, RMIT University, Bundoora, VIC 3083, Australia; yongmin.zhong@rmit.edu.au; 3 Department of Surgery, Monash University, Clayton, VIC 3800, Australia; Julian.Smith@monash.edu

**Keywords:** cytoskeleton, tensegrity, finite element modelling, local stiffness, atomic force microscope

## Abstract

The mechanical behaviour of adherent cells when subjected to the local indentation can be modelled via various approaches. Specifically, the tensegrity structure has been widely used in describing the organization of discrete intracellular cytoskeletal components, including microtubules (MTs) and microfilaments. The establishment of a tensegrity model for adherent cells has generally been done empirically, without a mathematically demonstrated methodology. In this study, a rotationally symmetric prism-shaped tensegrity structure is introduced, and it forms the basis of the proposed multi-level tensegrity model. The modelling approach utilizes the force density method to mathematically assure self-equilibrium. The proposed multi-level tensegrity model was developed by densely distributing the fundamental tensegrity structure in the intracellular space. In order to characterize the mechanical behaviour of the adherent cell during the atomic force microscopy (AFM) indentation with large deformation, an integrated model coupling the multi-level tensegrity model with a hyperelastic model was also established and applied. The coefficient of determination between the computational force-distance (F-D) curve and the experimental F-D curve was found to be at 0.977 in the integrated model on average. In the simulation range, along with the increase in the overall deformation, the local stiffness contributed by the cytoskeletal components decreased from 75% to 45%, while the contribution from the hyperelastic components increased correspondingly.

## 1. Introduction

In single cell microinjection, capillary pressure microinjection (CPM) systems are widely utilized to mechanically penetrate the cell membrane [1]. In the process of indenting large spherical cells, the interaction force can be detected by piezoelectric force sensors, enabling force-feedback control to be performed [2,3]. Recent investigations into the micropositioning of samples has demonstrated accurate and efficient placement on micrometre and nanometre scales [4,5,6,7]. Image processing algorithms have also been adopted to trace the deformation of the indented cells in order to improve the automated microinjection of single cells [8,9,10]. However, force sensors in CPM systems cannot accurately detect the nanonewton-scale interaction force between the capillary and the adherent cells [11]. For indenting adherent cells, atomic force microscopy (AFM) was introduced [12], and the force-distance (F-D) curve in indentation was recorded [13,14]. Combining microfluidic system with nano-channelled AFM cantilevers, single cell microinjection was achieved on adherent cells using AFM [15].

Hertzian models and their extended forms have been traditionally used to describe the nano-indentation conducted by AFM probes in various shapes [16]. By approximating the experimental F-D curve measured by the AFM, the local elasticity of the adherent cells was characterized by these models, represented by Young’s modulus [17,18,19,20]. In the aforementioned models based on the contact mechanics theory, the indented object was assumed to be a half-space with homogeneous isotropic material properties, and the deformation in the nano-indentation was required to be insignificant in comparison to the size of the object. However, biological cells are composed of multiple layers of continuum components such as the cell membrane, the actin cortex, the cytoplasm and the nucleus, as well as multiple discrete cytoskeletal components such as the microtubules (MTs), the actin filaments and the intermediate filaments [21]. Moreover, during AFM microinjection, the overall deformation of the cell could be too large for the half-space assumption in the Hertzian models to be valid. Hence, the Hertzian model is insufficient to characterize the mechanical response of adherent cells during AFM indentation with large cellular deformation.

To increase the accuracy of cell modelling, continuum models based on various non-linear elastic theories have been established. Initially, single layer membrane hyperelastic models were established by assuming the living biological cell as a fluid-filled entity enclosed by a layer of hyperelastic membrane, and different geometrical properties were introduced for different forms of the cell [22,23,24]. This single layer membrane hyperelastic model is capable of simulating comprehensive mechanical responses of the cell, including the overall deformation, the strain-stress relationship within the membrane and the force-distance relationship [22,23,24]. For a more realistic description of the cell, multi-layer hyperelastic models were also established by separately defining the material properties of the nucleus, cytoplasm and cell membrane of the cell, which are the continuum components [25,26]. Finite element method (FEM) has been widely used to resolve the increased complexity of the multi-layer hyperelastic model [25,26,27]. Apart from nano-indentation, various forms of mechanical stimuli, such as indentation, compression and elongation, were simulated by the hyperelastic models [22,23,24,25,26,27]. Many other non-linear elastic theories, such as viscoelastic and poroelastic, have also been investigated to characterize the time dependent stress-relaxation behaviour of the cell during nano-indentation [28]. Because of the versatility of the multi-layer hyperelastic model in describing the mechanical response of living cells under mechanical stimuli [25,26,27], it is adopted in this paper to simulate the continuum components of the cell during central indentation.

The observation and investigation of the intracellular microstructure in eukaryotic cells proved that the interconnected framework of discrete cytoskeleton plays a critical roles in cell’s mechanics [29,30]. Cytoskeletal components were shown to be pre-stressed, which led to the development of the self-equilibrium tensegrity structure for describing the cytoskeleton framework [31,32,33]. Therefore, tensegrity models were established by the microstructural approach. Within the cytoskeleton, the MTs resist the compression, while the actin filaments and intermediate filaments resist tension only [29]. In most adherent cells, actin filaments were observed to organise into higher-order structure as thick actin filaments bundles (AFBs) [34]. For spherical suspended cells, structures with abundant interconnected struts and cables can be developed using fundamental tensegrity models, which achieves mathematical self-equilibrium [35,36]. However, because of the morphology of adherent cells, the establishment of tensegrity model was usually empirical, without any mathematically demonstrated methodology [25,37].

In this paper, in order to constitute a mathematically demonstrated modelling method for the cytoskeleton framework in roundish adherent cells, a multi-level tensegrity model is established by distributing a group of rotationally symmetric prism-shaped tensegrity structures within the contour of the adherent cell. Although the fundamental structure of the proposed model is a single tensegrity structure with only twelve cytoskeletal components, by assigning the structures into multiple levels, the high density of cytoskeletal components is accurately replicated. The initial strains assigned to the cytoskeletal components are validated by the force density method, which assures the self-equilibrium of the structures. To evaluate the proposed model, human embryonic kidney 293 (HEK-293) cell was scanned and indented by the AFM system. Using FEM, the multi-level tensegrity model, a hyperelastic model, as well as an integrated model combining both the tensegrity model and the hyperelastic model have been established to characterize mechanical behaviour of the HEK-293 cells during AFM indentation. The proposed models have been validated by evaluating the similarity between the experimental F-D curves with the computational results, and the performances of the models are compared. Furthermore, an indentation distance-dependent local stiffness study was conducted to investigate the contribution from different components in the model to the local stiffness of the HEK-293 in the AFM indentation.

## 2. Modelling Methods

### 2.1. The Multi-Level Tensegrity Model

In adherent cells, thick AFBs with pre-existing tension are localized at the cell periphery beneath the plasma membrane, and the actin bundle framework plays a critical role in transmitting intracellular forces between the focal adhesion site. Meanwhile, thin intermediate filaments are extended from the nucleus to the cell periphery. Both the actin filaments and the intermediate filaments sustain tension during the cellular deformation [31]. On the other hand, pre-compressed MTs are widely distributed with the cytoplasm space. The MTs are hollow tubes with a higher second moment of inertia, which enables them to withstand compression more efficiently [34]. The aforementioned cytoskeletal components are mutually anchored at the end point such that the cell remains mechanically balanced.

To establish the tensegrity model of the cytoskeleton framework, two simplifications were applied. Firstly, in the cytoskeleton structure of an adherent cell, the contribution from intermediate filaments on the overall cellular stiffness is insignificant in comparison to the contribution from the thick AFBs and the MTs [25,34]. Hence, the intermediate filaments framework was neglected by the tensegrity model in this paper. Secondly, due to the nature of withstanding compression force, the MTs are observed to be buckling in adherent cells. In order to simplify the calculation, it was assumed that the compressing force generated by the MT obeys linear Hooke’s law against the distance between the nodes. Therefore, MTs in the proposed model are visualized to be linear throughout deformation in this study. The Young’s modulus of the MT was hence evaluated empirically, based on the geometry and the bending spring constant, which was experimentally determined [38]. Young’s modulus was also directly applied as the material property of the AFB. The estimated elastic and geometric properties of the cytoskeletal components are summarized in Table 1.

**Figure 1:**
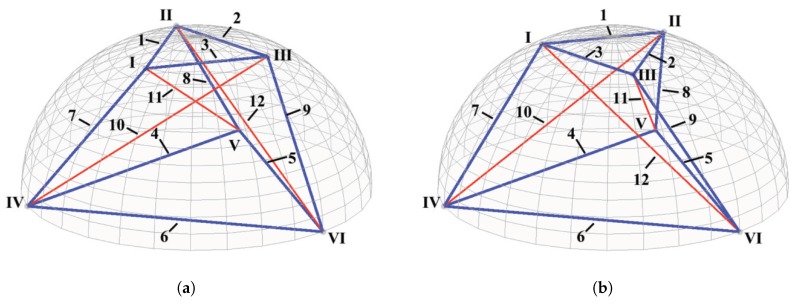
A rotationally symmetric prism-shaped tensegrity structure within a half-ellipsoid contour: (**a**) left-handed form (**b**) right-handed form.

Living HEK-293 cells adhering to a substrate are approximately round in the top view and form a half-ellipse from the side view. The form of cytoskeleton framework in a HEK-293 cell can thus be modelled by rotationally symmetric prism-shaped tensegrity structures with nodes on the contour of the cell membrane. The particular prism-shaped tensegrity structure is illustrated in Figure 1, which consists of six nodes and twelve members. In this structure, the members represent the cytoskeletal components, and the nodes represent the junction between the cytoskeletal components. Specifically, members 1 to 9 are the pre-tensioned AFBs, while members 10 to 12 are the pre-compressed MTs.

In the tensegrity structure, nodes I to III are the free nodes attached to the plasma membrane, whereas nodes IV to VI are the fixed nodes anchored to the substrate surface together with the membrane. Based on the chirality of the tensegrity structure, the structure is feasible to be performed in the left-handed form (Figure 1a) or the right-handed form (Figure 1b). In this case, the free regular triangle I-II-III is parallel to the fixed regular triangle IV-V-VI, with the angle of rotation from the top view equalling π/3 (left-handed form) or -π/3 (right-handed form).

It has been proved that, pre-existing strain can be found in the cytoskeletal components in living cells without external stimuli. Therefore, to assure the self-equilibrium of the tensegrity structure, the force density method [39] was applied to discover the proper initial strain assigned to the cytoskeletal members. In the pre-stressed structure, the initial force density $q_{i}$ in the member *i* can be calculated by:

(1) $$q_{i}=\frac{\epsilon_{ini}E_{i}A_{i}}{l_{i}},$$

where in each member, $\epsilon_{ini}$ is the initial strain, $E_{i}$ is the Young’s modulus, $A_{i}$ is the original cross-sectional area, and $l_{i}$ is the pre-stressed length.

Regardless of the position of the nodes and the geometrical parameters of the members, the connection relationship between the nodes and the members in the proposed tensegrity structure is affirmed. Hence, the topology of the rotationally symmetric prism-shaped tensegrity can be described by an incidence matrix $C\in R^{m\times n}$, where *m* is the number of members, and *n* is the number of nodes. Assuming nodes *i* and *j* (i<j) are connected by the member *k* in the structure, the value of $C_{\left(k,p\right)}$ in the incidence matrix is defined as:

(2) $$C_{\left(k,p\right)}=\left\{\begin{array}{ccc} 1 & \; & p=i\\ -1 & \; & p=j\\ 0 & \; & elsewhere. \end{array}\right.$$

As a result, the incident matrix $C_{L}$ and $C_{R}$ for the forms of the rotationally symmetric prism-shaped tensegrity as illustrated in Figure 1a,b are separately written as:

(3) $$\begin{array}{c} C_{L}=\left\{\begin{array}{cccccc} 1 & -1 & 0 & 0 & 0 & 0\\ 0 & 1 & -1 & 0 & 0 & 0\\ 1 & 0 & -1 & 0 & 0 & 0\\ 0 & 0 & 0 & 1 & -1 & 0\\ 0 & 0 & 0 & 0 & 1 & -1\\ 0 & 0 & 0 & 1 & 0 & -1\\ 1 & 0 & 0 & -1 & 0 & 0\\ 0 & 1 & 0 & 0 & -1 & 0\\ 0 & 0 & 1 & 0 & 0 & -1\\ 0 & 0 & 1 & -1 & 0 & 0\\ 1 & 0 & 0 & 0 & -1 & 0\\ 0 & 1 & 0 & 0 & 0 & -1 \end{array}\right\},C_{R}=\left\{\begin{array}{cccccc} 1 & -1 & 0 & 0 & 0 & 0\\ 0 & 1 & -1 & 0 & 0 & 0\\ 1 & 0 & -1 & 0 & 0 & 0\\ 0 & 0 & 0 & 1 & -1 & 0\\ 0 & 0 & 0 & 0 & 1 & -1\\ 0 & 0 & 0 & 1 & 0 & -1\\ 1 & 0 & 0 & -1 & 0 & 0\\ 0 & 1 & 0 & 0 & -1 & 0\\ 0 & 0 & 1 & 0 & 0 & -1\\ 0 & 1 & 0 & -1 & 0 & 0\\ 0 & 0 & 1 & 0 & -1 & 0\\ 1 & 0 & 0 & 0 & 0 & -1 \end{array}\right\} \end{array}$$

For the tensegrity, let [xyz] be the coordinates of the nodes in a Cartesian coordinate system. The nodal coordinates vectors x, y and z$\in R^{6\times 1}$, as the proposed three-dimensional structure has six nodes.

The equilibrium matrix *B* is developed as:

(4) $$B=\left\{\begin{array}{c} C^{⊤}diag\left(Cx\right)\\ C^{⊤}diag\left(Cy\right)\\ C^{⊤}diag\left(Cz\right) \end{array}\right\}$$

To assure the self-equilibrium of the tensegrity structure, the force density vector should fulfil the following equation:

(5) $$Bq=\left\{\begin{array}{c} C^{⊤}diag\left(Cx\right)\\ C^{⊤}diag\left(Cy\right)\\ C^{⊤}diag\left(Cz\right) \end{array}\right\}q=0,$$

where $q\in R^{12\times 1}$ is the force density vector. Consequently, the feasible force density vector q was found based on the null space of the equilibrium matrix *B* using numerical iteration method [39]. Additionally, force density vectors c•q can also result in a valid tensegrity structure, where {c|c>0,c∈R}.

The proposed tensegrity structure is composed by the free nodes surface I-II-III in parallel to the fixed nodes surface IV-V-VI. By changing the distance between the free nodes surface and the fixed nodes surface, a group of rotationally symmetric prism-shaped tensegrity structures can be established in accordance with the contour of the cell. The diameter of the HEK-293 cell is generally found to be roughly around 20 μm and the height is around 5 μm.

**Figure 2:**
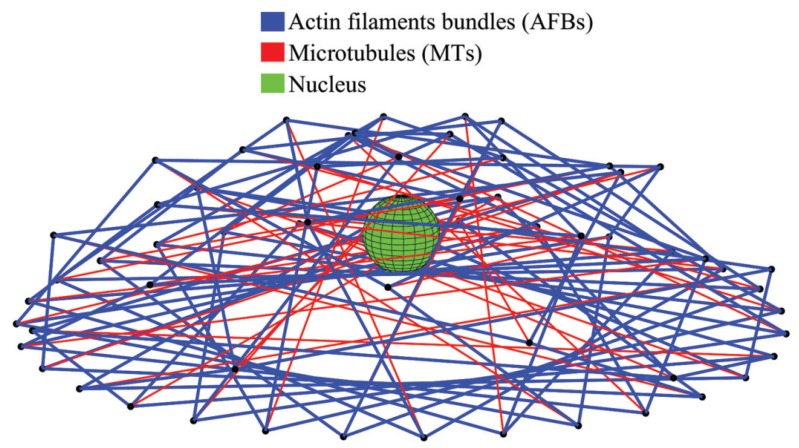
A ten-level tensegrity model within the HEK-293 cell’s contour.

Figure 2 illustrates a ten-level tensegrity model of the HEK-293 cell. Following the ascending order of the distance between the free nodes surface and the fixed nodes surface in the model, the tensegrity structures are numbered from level 1 to level 10. In the ten-level tensegrity model, totally 60 AFBs and 30 MTs are modelled as the cytoskeletal components. The free nodes surfaces are distributed in the intracellular space at different heights in the model. In this model, the odd numbered levels of the structure are in the left-handed form, while the even numbered levels are in the right-handed form. As a result, the potential rotational displacement would be countered within the adjacent levels of the tensegrity structure during the central indentation, and the cell can still approximately behave as an axis-symmetric entity during deformation. In the proposed ten-level tensegrity model of a HEK-293 cell, the lengths of the AFBs and MTs in each level are listed in Table 2.

According to the force density method, a feasible set of initial strain in different levels are listed in Table 3. The values of the initial strain can be changed proportionally within each level, with the structure remains self-equilibrium. Since the force density method has guaranteed the self-equilibrium of each level in this model, the ten-level tensegrity model is also self-balanced without external stimuli.

### 2.2. The Integrated Model

In order to accurately characterize the mechanical behaviour of the cell during indentation, apart from the discrete cytoskeleton framework, continuum components such as the cell membrane, the actin cortex, the cytoplasm and the nucleus have to be considered. An integrated model of HEK-293 cell combining the ten-level tensegrity model and the hyperelastic model was established by FEM in ANSYS (ANSYS, Inc., Canonsburg, PA). The hyperelastic model is a half ellipsoid adhering to an infinite rigid surface, with the height of 5 μm and the diameter of 10 μm. Firstly, the cell membrane and the actin cortex of the cell are together defined as a layer of 0.05 μm thickness membrane enclosing the cell. In order to maintain the geometrical shape of the biological cell, the membrane’s stiffness is significantly higher than the intracellular components. The nucleus is defined as a sphere with a 2 μm diameter, and its centre is located at the centre of the cell from the top view, 2 μm above the substrate. Neo-Hookean material has been widely adopted to describe the strain-stress relationship in the deformed hyperelastic components in biological cells [22,23,25], with the shear modulus providing the only material constant. The estimated shear modulus of the components in the multi-layer hyperelastic model are listed in Table 4. The AFM probe and the petri-dish surface were assumed to be rigid, as they have a significantly higher stiffness compared to that of the biological cells. In the tensegrity structure, LINK 180 element is selected to describe the cytoskeletal components within the FEA model, because they are uniaxial tension-compression element without bending, which is matches the assumptions of the MTs and AFBs in the model.

In the FEM model, a ’bonded’ contact was applied to the surfaces between the hyperelastic components, as well as the surfaces between the AFM probe and the membrane. Although in the nano-indentation models, the friction situation between the probe and the membrane significantly influences on the reaction force [40], during large deformation, the contact can be simplified to a ’bonded’ mode. In order to couple the hyperelastic model and the tensegrity model, a ’bonded’ contact was established between the cell membrane and the nodes in the tensegrity structure. Except the shared nodes on the membrane and the tensegrity structure, the contact within the cytoskeletal components, as well as the contact between the cytoskeletal components and the hyperelastic component, were neglected. As a result, the proposed ten-level tensegrity model and the hyperelastic model were coupled as the integrated model.

**Figure 3:**
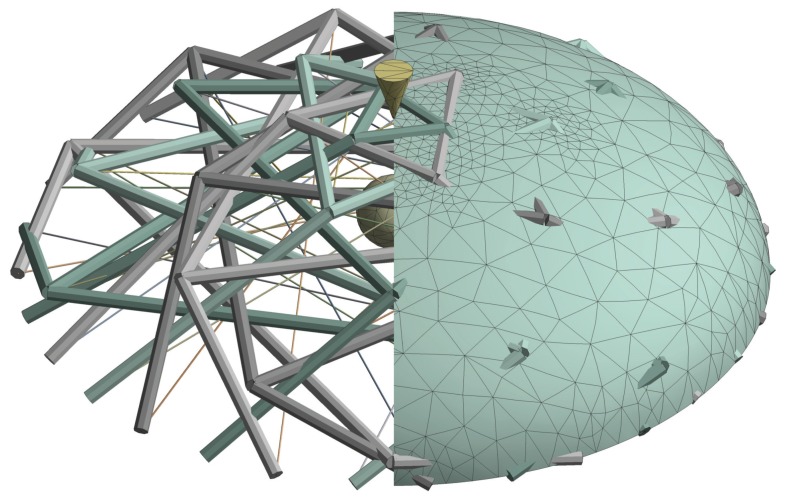
The integrated model of an HEK-293 cell indented by a rigid probe.

Figure 3 illustrates the integrated model established by FEM, exposing the internal tensegrity structure and the nucleus. Because the surface IV-V-VI is the fixed to petri-dish together with the membrane, no deformation occurs in the members 4–6 of the tensegrity structures. Hence, the AFBs numbered 4–6 in all levels were not modelled. Since the structure of the ten-level tensegrity model is not geometrically axis-symmetric, the cell was completely modelled. The total number of the finite elements in the integrated model is 10,864. With the establishment of the integrated model, the overall deformation of the cell at different indentation distances was able to be simulated, coupling the continuum hyperelastic components in the hyperelastic model and the discrete cytoskeletal components in the ten-level tensegrity model.

### 2.3. Central Indentation Simulation and the F-D Curve Similarity

In the integrated model, the deformation is determined by the contribution of the hyperelastic components and the cytoskeletal components simultaneously. However, FEM also enables the investigation of the behaviour of these components in isolation. As was previously discussed, it is assumed that AFBs withstand tension only while MTs withstand compression only, hence the interaction force attributed to the corresponding AFB (in compression) or MT (in tension) was not considered in the calculation. By aggregating the Z-axis force generated by the tensegrity structures, the F-D curve determined by the tensegrity model was also obtained.

To evaluate the accuracy of the models in characterizing the force-indentation distance relationship during central indentation of the HEK-293 cells, the experimental F-D curves measured by the AFM were compared to the computational F-D curves calculated by the models. In the hyperelastic model, the shear modulus of the membrane is considered to be the characteristic parameter that dominates the behaviour of the cell, hence it was used as the fitting variable in that part of the model. Similarly, by proportionally changing the values of the estimated Young’s modulus of the cytoskeletal components in Table 1, a bunch of computational F-D curves were generated by the tensegrity model. In the integrated model, the Young’s modulus of the cytoskeletal components was fixed, and the shear modulus of the membrane was adjusted to generate the F-D curves.

As a result, a group of computational F-D curves calculated by the models adopting different values of the fitting variables were obtained. The similarity between an experimental F-D curve and a computational F-D curve was quantitatively evaluated by coefficient of determination ($R^{2}$), where the higher $R^{2}$ indicates the higher similarity. Comparing the highest $R^{2}$ obtained, the performances of the hyperelastic model based on continuum mechanics, the ten-level tensegrity model using cytoskeletal approach, and the integrated model were investigated. The material properties resulting in the most similar F-D curve in the integrated model are considered as the parameters in the control model.

### 2.4. Local Stiffness Analysis

Local stiffness analysis was conducted to reveal the contribution of the different mechanical components in the integrated model to the stiffness of the cells. Because of the non-linearity of the F-D relationship in cell indentation [13], the local stiffness of the cell should be an indentation distance-dependent value. The local stiffness k(δ) at the particular indentation distance δ(i) is defined as:

(6) $$k\left(\delta \right)=\frac{dF}{d\delta }\approx \frac{F(i)-F(i-1)}{\delta (i)-\delta (i-1)},$$

where F(i) and δ(i) is the corresponding indentation force and indentation distance at the sample point on the F-D curve, respectively. As a result, a stiffness-indentation curve can be calculated by the control model with the most similar computational result. The sensitivity study was conducted by independently examining reaction force responded by the hyperelastic and cytoskeletal components in the control model, where the contribution to the local stiffness of the cell from each component was analysed.

## 3. Experimental Facilities and Methods

### 3.1. Cell Preparation

The indentation was conducted on HEK-293 cell line (ATCC, Manassas, VA, USA) in a μ-35 mm low petri-dish (IBIDI, Martinsried, Germany) with the culture medium consisting of 100% Phosphate-buffered Saline (PBS). Before the indentation, the HEK-293 cells were cultured and incubated in 100% Dulbecco’s Modified Eagle Medium, 37 ${}^{\circ }C$, 5% CO_2_ environment for 20 h. Before the measurement, the cells and the petri-dish were washed in PBS three times.

### 3.2. AFM System

The experimentation was performed using FlexAFM (Nanosurf, Liestal, Switzerland) system through the Nanosurf C3000 (Nanosurf, Liestal, Switzerland) software. The AFM probe used in the imaging and indentation was ANSCM-PC (APPNANO, Silicon Valley, CA, USA). The nominal spring constant of the cantilever is 0.2 N/m. The nominal frequency of the cantilever is 12kHz. The tip radius of the probe is 0.03 μm. The tip height of the probe is 14 μm to 16 μm, which is significantly higher than a normal HEK-293 cell. The cantilever is coated with Pt/Ir, which enables the long-term measurement in liquid environment. The probe is designed to be used in the static force mode.

### 3.3. Central Indentation

**Figure 4:**
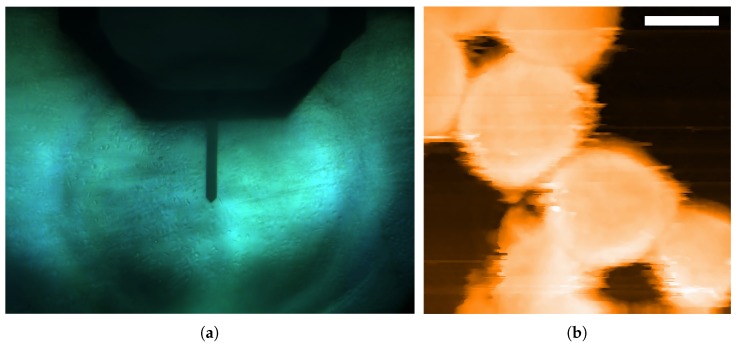
HKE-293 cells in PBS environment: (**a**) The experimental environment for the central indentation of HEK-293 cells (**b**) HEK-293 cells imaged by the AFM, Scale bar: 10 μm.

The scan and indentation of HEK-293 cells were conducted in the PBS environment to keep the HEK-293 cells active. Figure 4a shows the optical top view of the typical experimental environment in this study, and the picture is taken by FlexAFM video camera (Nanosurf, Liestal, Switzerland) attached to the AFM system. First of all, the actual spring constant of the cantilever needs to be calibrated, since the static force mode would be adopted. The calibration was performed using the Nanosurf C3000’s thermal tuning mode and repeated five times. The average value of the spring constant and the Q factor were introduced as the properties of the cantilever.

Before the indentation, the location and geometry of the cells need to be confirmed in the image mode. Due to the size of the HEK-293 cells, the adhesion force of adherent cells are very small at around 100 nN [41,42]. Hence, in order to decrease the impact from the lateral force on the adhesion of the target cells, the Z-axis controller force set point was set to be 0.1 nN to 0.3 nN, and the scanning speed was set as 12.5 μm/s in the first scan. As illustrated in Figure 4b, the cells were scanned in static force mode by the AFM system, and the diameter of the cells from the top view, as well as the overall height information of the scanned area, can be obtained.

Subsequently, the location of the central point of the cells were manually decided, where the central indentations were performed in the spectroscopy mode. In the spectroscopy mode, the AFM probe started the indentation from 0.5 μm above the highest scanned point in the area, aiming at the centre of each cell successively. The Z-axis of the AFM scan-head worked in the fixed stop value mode. Once the interaction force reaches 15.0 nN, the probed was paused for 1.0 s and was then fully retracted. The indentation speed was set as 0.5 μm/s.

**Figure 5:**
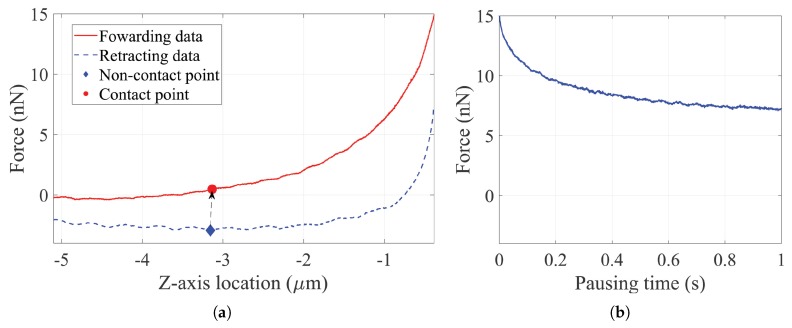
Original experimental data in the central indentation on a HEK-293 cell measured by the AFM: (**a**) the forwarding data and the retracting data (**b**) the pausing time data.

The process was repeated on fourteen individual cells in a short period of time. The experimental F-D curves were thus measured from the cells in the same life stage. Figure 5 presents a set of original data measured in the central indentation on a HEK-293 cell, including the probe forwarding data, probe retracting data and pausing time data. The horizontal axis data is denoted the Z-axis location of the AFM probe, and a negative value indicates the location is above the petri-dish surface referenced by the previous scanning result. The stress-relaxation behaviour of the deformed biological cells causes the discrepancy of the detected forces before and after the pause [28].

### 3.4. Data Process

In order to quantitatively analyse the performance of the theoretical models in the following sections, experimental F-D curves measured by the AFM need to be processed. First of all, the contact point (CP) is the particular force-distance point where the probe starts to contact the surface of the cell. Because of the low rigidity of biological cells, an error is inevitable in identifying the CP. However, an error in the CP selection will not significantly influence the results of the force approximation [16]. Due to the adhesion force between the probe and the cell, the CP selection references the minimal force point ($z_{np}$, $F_{np}$) with the negative force value on the retracting data as the non-contact point (NP) [40]. The cantilever deflection at the NP can be calculated by:

(7) $$\Delta d^{\prime }=\frac{F_{np}}{k_{c}}$$

and the Z-axis location of the CP $z_{0}$ on the forwarding data can be found at:

(8) $$z_{0}=z_{np}-\Delta d^{\prime }.$$

Figure 5a illustrated the relationship between the NP and CP. With the CP selected, the experimental F-D curves were trimmed from the zero point to the maximum force point, or the point before the penetration point [13,14] if possible. In addition, the length of the trimmed F-D curve was larger than 2 μm, so that the final length of the smoothed F-D curve would not be smaller than 2 μm.

To compensate the system error in Z-axis caused by the cantilever deflection, the actual indentation distance is calculated using the spring constant of the cantilever. In the trimmed curve, the Z-axis value ranges from $z=z_{0}$ to $z=z_{max}$, while the force value ranges from $F_{0}$ to $F_{max}$. Since the deflection of the cantilever Δd can be denoted by:

(9) $$\Delta d=\frac{F}{k_{c}},$$

where *F* is the detected force, and $k_{c}$ is the spring constant of the AFM cantilever. Therefore, the actual indentation distance δ corresponding to *F* can be calculated by:

(10) $$\delta =\left(z-z_{0}\right)-\left(\Delta d-\Delta d_{0}\right)=z-\left(\frac{F}{k_{c}}-\frac{F_{0}}{k_{c}}\right),$$

where $F_{0}$ is the force at the CP, and the $\Delta d_{0}$ means the cantilever deflection at the CP. As a result, the F-z curve was transferred to the F-δ curve with δ ranging from zero to $z_{max}-z_{0}$ and *F* ranging from zero to $F_{max}-F_{0}$. In the subsequent content, the term F-D curve refers to the F-δ curve unless mentioned. By these methods, the Z-axis coordinate decreasing from $z_{0}$ to $z_{max}$ in the experimental F-D curve was transferred to the indentation distance δ increasing from zero to $\delta_{max}$, and the effect of cantilever’s deflection during indentation is eliminated.

To remove the environmental noise during the experimentation, the experimental F-D curves were smoothed using the moving average filter. The smoothed F-D curves were again trimmed from the CP to the δ= 2.0 μm point to guarantee the consistent length of all experimental data, which can be utilized to evaluate the proposed model.

## 4. Results

In the simulation, the indentation distance increased from 0 μm to 2 μm from the centre of the cell model in the top view. The mechanical features of the components used in the simulation are listed in Table 1, Table 2, Table 3 and Table 4, unless especially mentioned.

### 4.1. Mechanical Response of the Hyperelastic Components

**Figure 6:**
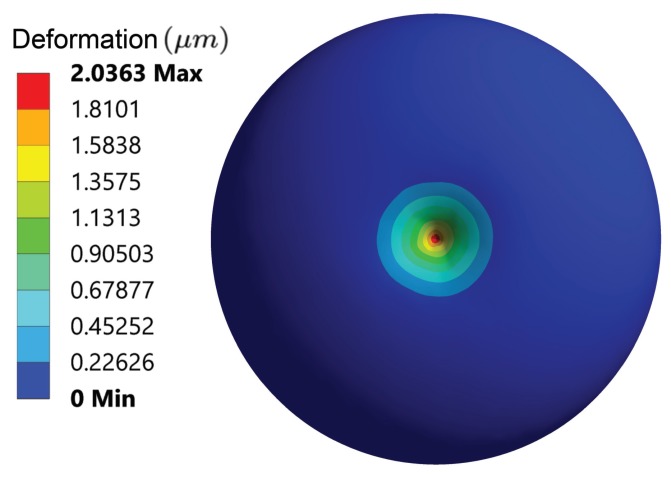
The total deformation of the hyperelastic components from the top view when indentation distance reaches 2 μm.

**Figure 7:**
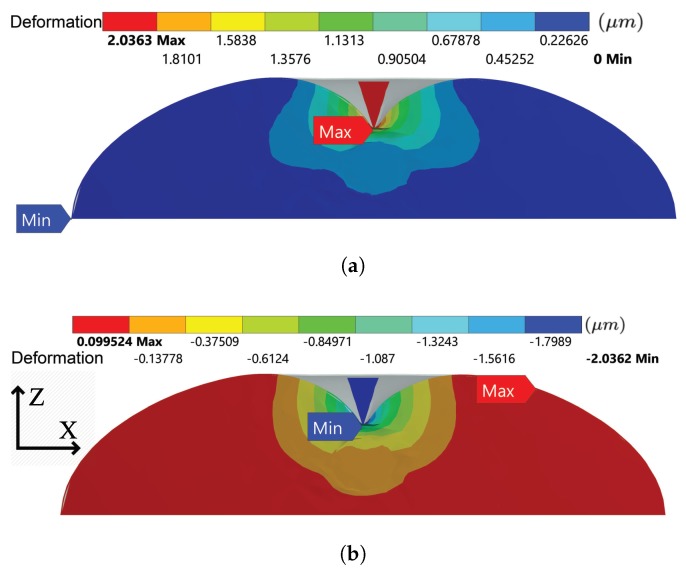
The deformation of the hyperelastic components in the representative cross section when indentation distance reaches 2 μm: (**a**) total deformation (**b**) Z-axis deformation.

For the hyperelastic components in the integrated model, the total deformation from the top view externally is illustrated in Figure 6. The distribution of the total deformation suggests that although the ten-level tensegrity model is not axis-symmetric, by introducing the tensegrity structures with different chirality, the mechanical behaviour of the coupled hyperelastic components during the central indentation is still approximately axis-symmetric. The total deformation of the internal hyperelastic components is illustrated in Figure 7a, and Figure 7b depicts the directional deformation at Z-axis. The approximate axis-symmetric behaviour of the hyperelastic components assures that the results can be generalise to the global response of the cell. The distributions of the absolute value of the total deformation and directional deformation in Figure 7 display high similarity, which suggests that the Z-axis deformation is the dominant directional deformation in the hyperelastic components during the central indentation.

### 4.2. Mechanical Responses of the Cytoskeletal Components

**Figure 8:**
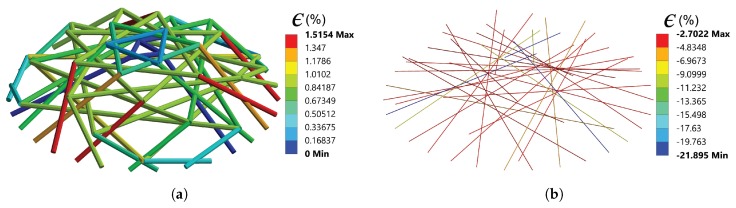
The axial strain in the cytoskeletal components when indentation distance reaches 2 μm: (**a**) distribution of axial strain in AFBs (**b**) distribution of axial strain in MTs.

Figure 8 shows the strain in the deformed AFBs and MTs in the ten-level tensegrity structure, when the indentation distance reaches 2 μm. In each level of the tensegrity model, nodes IV to VI are fixed to the substrate, and the AFBs in the fixed surface were thus not modelled. The axial strain ϵ is defined as the sum of the initial strain and the strain caused by the central indentation:

(11) $$\epsilon =\frac{L\left(1+\epsilon_{ini}\right)-L_{pre}}{L_{pre}}\times 100\%,$$

where $\epsilon_{ini}$ is the initial strain, *L* is the current length of the cytoskeletal component during the indentation, and $L_{pre}$ is the pre-stressed length of the cytoskeletal component before the indentation.

Since there are 10 levels of tensegrity structures with 90 cytoskeletal components modelled, the particular component is numbered after the level number and the member number. For example, AFB 2.1 denotes the member 1 in level 2 (which is an AFB) in the following sections. In the central indentation simulation, except for AFBs 10.7 to 10.9, the other 57 AFBs end up withstanding tension at the largest deformation. In comparison to the initial strain, except for the AFBs in level 1 (the axial strain increases from 1.00% to 1.04%), the majority of the AFBs in the free nodes surface (AFBs x.1 to x.3) experience the reduction of tension after deformation. The axial strain especially reduces remarkably in the AFBs x.1 to x.3 in levels 8–10, whose free nodes are close to the indentation point. In contrast, in levels 3–8, significant growth of strain is observed in AFBs x.7 to x.9. The highest strain occurs in AFBs 7.7 to 7.9, which is the level adjacent to the maximum Z-axis deformation. Since the AFBs were defined as LINK 180 element that bears tension only, no axial strain is held by the AFBs 10.7 to 10.9 when δ reaches 2 μm, as the compression in the aforementioned AFBs exceeds the initial tension during the indentation.

In the MTs, a significant increase in axial strain appeared in the levels close to the indentation point, with the highest strain increases from −5.57% to −21.90% in level 10. In the rest of the tensegrity structures, the initial compression in the MTs was relieved to some extent, while all MTs remained compressed throughout the indentation test.

**Figure 9:**
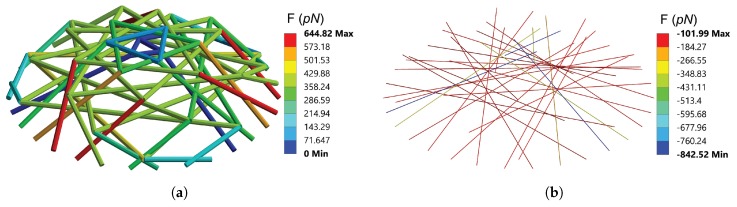
The axial force in the cytoskeletal components when indentation distance reaches 2 μm: (**a**) distribution of axial force in AFBs (**b**) distribution of axial force in MTs.

Figure 9 presents the axial force in the cytoskeletal components when the indentation distance is 2 μm. Forces within pN range are observed. Tensile forces can be found in most of the AFBs, except AFBs 10.1 to 10.3. All MTs in the tensegrity structures were resisting compression force at the 2 μm indentation distance.

### 4.3. Evaluating the Models’ Accuracy

**Figure 10:**
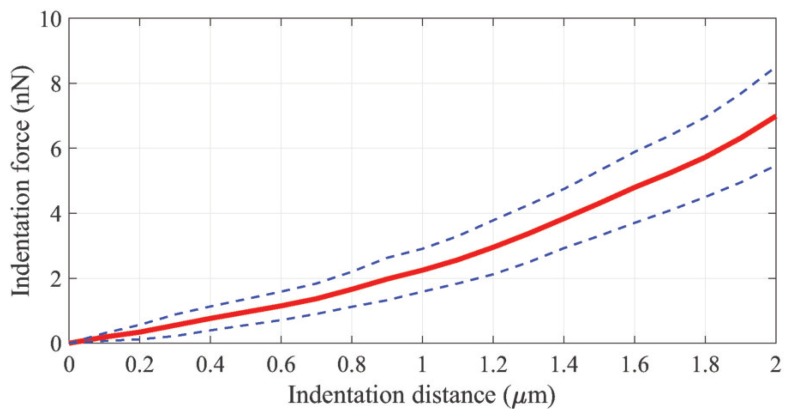
The average pre-processed experimental F-D curves. Dashed lines present the standard deviation of the forces.

In the F-D curves, points from 0 μm to 2 μm at the 0.1 μm increments were sampled to quantitatively evaluate the similarity between the computational and experimental F-D curves by $R^{2}$. Figure 10 depicts the averaged pre-processed experimental F-D curve derived from the fourteen experimental F-D curves measured by the AFM, together with the ± standard deviation (SD).

**Figure 11:**
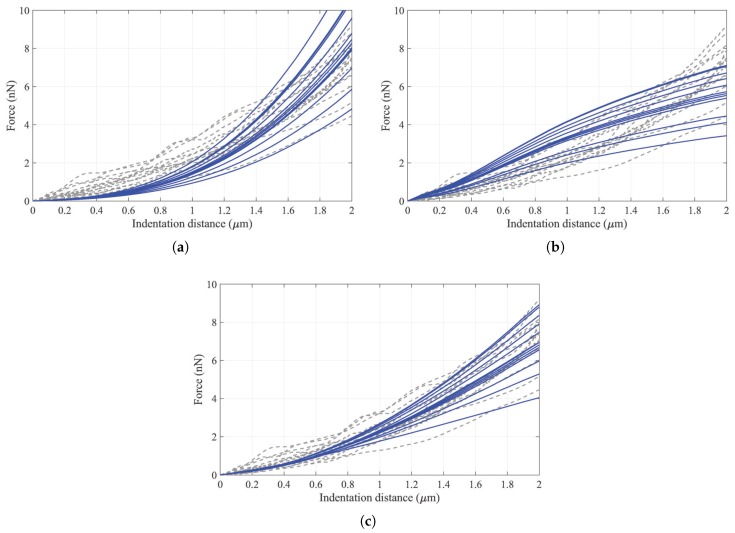
The highest $R^{2}$ results provided by the (**a**) hyperelastic model (**b**) ten-level tensegrity model (**c**) integrated model; The dash lines are the experimental F-D curves, and the solid lines are corresponding computational F-D curves with the best similarity.

**Figure 12:**
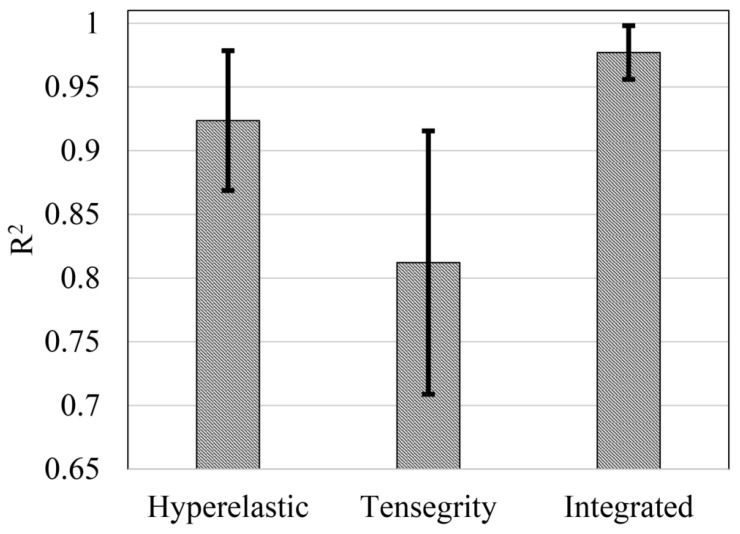
Distributions of the $R^{2}$ between the most similar computational F-D curves and the experimental F-D curves.

Figure 11 presents the results with the highest similarity in the computational F-D curves calculated by the hyperelastic model, the ten-level tensegrity model, and the integrated model, to the experimental F-D curves. In addition, the average values and the standard deviation of $R^{2}$ achieved by the models are presented in Figure 12.

The best similarity of computational F-D curves was realized by the integrated model, with the average $R^{2}=0.977$. For the non-integrated models, the hyperelastic model is vastly superior to the tensegrity model, with the average $R^{2}=0.924$ compared to $R^{2}=0.812$. Furthermore, the standard deviation indicates the consistency of the model in simulating the F-D curve of the cell. The hyperelastic model and the tensegrity model present the standard deviation at ±0.055 and ±0.103, respectively. While the integrated model yields the smallest standard deviation at ±0.021.

By approximating the experimental forces by the numerical models, the material properties, as the fitting variable in the models, were estimated as given in Table 5. In the hyperelastic model, the shear modulus $C_{Mem}$ of the cell membrane was estimated at (0.96±0.22) MPa, and the $C_{Mem}$ in the integrated model is slightly smaller at (0.67±0.20) MPa. In the tensegrity model, the Young’s modulus of the AFBs and the MTs are $E_{AFB}$= (1.97±0.38) MPa and $E_{MT}$= (290.00±55.69) MPa, respectively.

### 4.4. Local Stiffness Analysis

**Figure 13:**
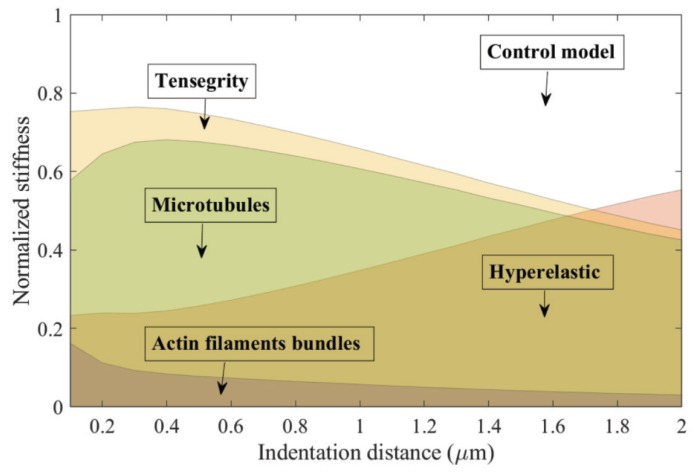
The average normalized stiffness attributed to different components in the integrated model.

Figure 13 depicts the results of the average local stiffness of the cell, clarifying the contribution from different components in the integrated control model. The stiffness-indentation distance curve of each component was normalized with respect to the stiffness of the control model.

The normalized stiffness curves show that, at small indentation, the ten-level tensegrity structure dominates the mechanical behaviour of the cell, contributing approximately 75% of the local stiffness. As deformation increases, the percentage of the local stiffness attributed to the hyperelastic components correspondingly increases. The hyperelastic components dominate the response when the indentation distance is over 1.7 μm, on average. Within the tensegrity structure, the local stiffness attributed to the AFBs is overwhelmed by the contribution from the MTs during the indentation, regardless of the overall deformation.

## 5. Discussion

In the central indentation simulation, the cellular structure was mechanically withstanding the mechanical load in compression. Therefore, mechanical response from the MTs in the cytoskeleton should dominates the behaviour of the cell. FEM simulation results demonstrated that, more significant axial strain was observed in the MTs rather than the AFBs. The distribution of the axial force leads to the similar results that higher axial forces were found in the MTs that in the form of compression. For the hyperelastic components, significant deformation can only be found in the area adjacent to the indentation point. Similar behaviour is exhibited by the MTs in the simulation, whereas, in the AFBs, the largest tensile strain and force were observed in level 7, whose free surface nodes are close to the location that the maximum positive Z-axis deformation was found. The distribution of the axial strain and force in the cytoskeletal components proves that the mechanical response of the adherent cell in the central indentation is a global behaviour. During the indentation, with the assumed initial strain allocated, all MTs and the majority of AFBs in the integrated model keep resisting the compression or tension only, separately. The pN range axial forces in the cytoskeletal components would not exceed the load that a single MT [43] or an AFB [31] can sustain. Therefore, the proposed ten-level tensegrity structure can resist large deformation in indentation, without significant mechanical failure in the cytoskeletal components.

Computational F-D curves obtained by the ten-level tensegrity model and the hyperelastic model display significant non-linearity with the indentation distance increasing. However, the computational F-D curves of the ten-level tensegrity model act as convex functions in the large deformation region, which is in contradiction to the experimental results measured by the AFM. Although the computational F-D curves of the hyperelastic model develops as concave functions, the non-linearity is constantly overestimated by the model compared to the experimental results. By approximating the experimental F-D curves with the integrated model results, the highest similarity results among the models were found. The integrated model achieves not only the highest average $R^{2}$, but also the smallest standard deviation of $R^{2}$. Therefore, the accuracy and the consistency of the proposed integrated model in terms of simulating the F-D curve in AFM indentation within the experimental range were demonstrated. In addition, the calibrated shear modulus of the cell membrane is located in the range of the results obtained by the previous studies [22,25], which demonstrates the validity of the proposed integrated model.

Both the cytoskeletal components and the hyperelastic components in a biological cell play crucial roles in responding to external stimuli. Meanwhile, the non-linear force response during the indentation determines that the constant stiffness analysis [19] method inadequately describes the local stiffness contribution to the cell from individual components. Figure 13 indicates that at the small deformation stage of the indentation, the ten-level tensegrity structure contribute more than 70% of the local stiffness to the cell. On the other hand, the non-linear nature of Neo-Hookean material leads to the rapid increase in the stiffness attributed to the hyperelastic components as the deformation increases. Consequently, the contribution from the hyperelastic components to the overall stiffness exceeds the counterparts in the tensegrity structure in the large deformation region. Among the cytoskeletal components, the overall compression is mainly accommodated by the MTs, and the initial tension within the AFBs is partially countered by the compression tendency during the indentation. The dominating contribution from the MTs to the local stiffness within the cytoskeletal components is hence explained.

Notably, in a previous study, the strain stiffening behaviour was observed in the micro-filaments when the tension ratio was higher than 30% [44]. However, in the proposed model, the tension ratio was significantly lower than the value which would cause the strain stiffening behaviour, while the material properties of the cytoskeletal components and the hyperelastic components were set as constants. Therefore, the increase in local stiffness during the indentation is attributable to the cellular morphology and the organization of the tensegrity structure as a global behaviour, which makes the possible strain stiffening behaviour of any single component in the proposed model irrelevant.

## 6. Conclusions

In this paper, a ten-level tensegrity model characterizing mechanical response of the HEK-293 cell in AFM indentation has been established. The self-equilibrium of the tensegrity structure is guaranteed by the force density method. The proposed model also reflects the denseness of cytoskeletal components in the intracellular space, by modelling 60 AFBs and 30 MTs in total. In the central indentation test, the combination of the ten-level tensegrity model and a hyperelastic model was demonstrated to accurately describe the non-linear force-deformation response characteristics. Although a large deformation was applied to the cells, the axial deformation of the single MT or AFB in the structure was relatively small. Hence, mechanical failure was not observed in the cytoskeletal components in the tensegrity structure. The experimentally observed non-linearity in the F-D curve suggests that, local stiffness is not a constant during indentation. The indentation distance-dependent stiffness analysis of the integrated model shows that, the cytoskeletal components dominate the local stiffness in the small deformation region. Whereas the local stiffness attributed to the hyperelastic components rapidly increases with deformation. Within the cytoskeletal components, MTs constantly dominate the cell stiffness throughout the indentation, while the contribution from the AFBs turns out to be insignificant in comparison. Overall, this study provided a generalized methodology for establishing a multi-level tensegrity model for roundish adherent cells, which characterize the mechanical function of the cytoskeleton framework. Furthermore, the normalized stiffness-indentation distance curve reveals new insights into the deformation-dependent local stiffness analysis during cell indentation.

## Figures and Tables

**Table 1:** Estimated elastic and geometric properties of the cytoskeletal components.

Component	Young’s Modulus (Pa)	Poisson’s Ratio	Diameter (nm)
Actin filiaments bundle [25]	$3.4\times 10^{5}$	0.3	200
Microtubule [38]	$5.0\times 10^{7}$	0.3	(outer/inner) 23/17

**Table 2:** The pre-stressed length of the cytoskeletal components, with the unit of μm.

Level No.	AFB 1∼3	AFB 4∼6	AFB 7∼9	MT 10∼12
1	17.20	17.32	5.21	19.26
2	16.67	17.32	5.28	19.00
3	15.80	17.32	5.43	18.59
4	14.61	17.32	5.69	18.02
5	13.12	17.32	6.07	17.30
6	11.37	17.32	6.61	16.46
7	9.38	17.32	7.30	15.52
8	7.21	17.32	8.12	14.49
9	4.88	17.32	9.06	13.41
10	2.46	17.32	10.09	12.29

**Table 3:** The assigned initial strain in the cytoskeletal components.

Level No.	AFB 1∼3	AFB 4∼6	AFB 7∼9	MT 10∼12
1	1.00%	0.99%	0.50%	−8.73%
2	1.00%	0.96%	0.53%	−8.64%
3	1.00%	0.91%	0.54%	−8.45%
4	1.00%	0.84%	0.57%	−8.20%
5	1.00%	0.76%	0.61%	−7.82%
6	1.00%	0.66%	0.70%	−7.44%
7	1.00%	0.54%	0.73%	−7.05%
8	1.00%	0.42%	0.81%	−6.58%
9	1.00%	0.28%	0.91%	−6.10%
10	1.00%	0.14%	1.01%	−5.57%

**Table 4:** Estimated shear modulus of the hyperelastic components.

Components	Shear Modulus (Pa)
Membrane [22]	$5.0\times 10^{5}$
Cytoplasm [25]	$1.7\times 10^{2}$
Nucleus [25]	$1.7\times 10^{3}$

**Table 5:** Material properties estimated in different modelling approaches.

Property	Hyperelastic	Tensegrity	Integrated
$C_{mem}$ (MPa)	0.96±0.29	/	0.67±0.20
$E_{AFB}$ (MPa)	/	1.97±0.38	0.34
$E_{MT}$ (MPa)	/	290.00±55.69	50.00
